# Understanding the Interplay between Wellness Motivation, Engagement, Satisfaction, and Destination Loyalty

**DOI:** 10.3390/bs14030239

**Published:** 2024-03-15

**Authors:** Young-joo Ahn, Katie Bokyun Kim

**Affiliations:** 1 Department of Hospitality and Tourism Management, Sejong University, Seoul 05006, Republic of Korea; yjahn@sejong.ac.kr; 2 Department of English, Cyber Hankuk University of Foreign Studies, Seoul 02450, Republic of Korea

**Keywords:** wellness tourism, wellness motivation, reflective engagement, experiential engagement, satisfaction, destination loyalty

## Abstract

Increased awareness of negative psychological symptoms and the negative impact of the pandemic has led to a rising demand for wellness-related travel experiences. There is a need for research on tourists’ experiential and reflective engagement in order to maximize positive outcomes such as overall satisfaction, positive WOM, and recommendations. These positive outcomes are crucial for attracting tourists and strengthening destinations’ brands. As there are few empirical studies, research on the effects of engagement on satisfaction and behavioral intentions is necessary. This study aimed to examine the relationships between wellness motivation, engagement, satisfaction, and destination loyalty among wellness tourists. It also aimed to examine the mediating effects of two engagement factors, experiential and reflective engagement, between wellness motivation and positive outcomes. A total of 319 respondents were used for the analysis, and structural equation modeling (SEM) was conducted. The results found that wellness motivation is composed of six wellness motivation components, namely physical motivation, transcendence, relaxation, social motivation, self-esteem, and escape, each representing first-order factors. Wellness motivation is positively associated with reflective and experiential engagement. Engagement positively affects satisfaction and destination loyalty. This study provides several implications, theoretically and practically.

## 1. Introduction

The popularity of wellness tourism has increased, driven by a growing focus on health and well-being, particularly in response to negative psychological effects such as stress, depression, and burnout following the COVID-19 pandemic [1]. Increased awareness of these negative psychological symptoms and the negative impact of the pandemic has led to a rising demand for wellness-related travel experiences that prioritize physical, mental, and emotional wellness [2]. The Global Wellness Institute (GWI) [1] highlighted the remarkable growth rate of 36% per year in wellness tourism since 2020. The GWI indicated that consumers are expected to spend on wellness, and the wellness industry will grow at an annual rate of 8.6%, reaching expenditures of USD 6.3 trillion at the end of 2024 and USD 8.5 trillion in 2027. South Korea, an emerging destination for medical and wellness tourism, holds significant growth potential, showing promising signs for the future [3]. Wellness destinations are attracting a diverse influx of tourists in search of holistic wellness treatments and immersive natural and cultural experiences [2,4].

Previous research on wellness tourism has explored wellness motivation, segments and profiles, wellness values, attributes, and wellness experiences [5,6,7,8,9,10,11,12,13]. Scholars have called for research on wellness motivation and engagement at wellness destinations, which are essential for achieving positive outcomes for sustainable destination management [14,15,16].

Even though there is an increasing demand for health and wellness tourism, only a handful of studies have been carried out on wellness motivation, engagement, and positive outcomes in different cultural contexts in hospitality and tourism. Moreover, there is a need for research on tourists’ experiential and reflective engagement in order to maximize positive outcomes such as overall satisfaction, positive WOM, and recommendations. These positive outcomes are crucial for attracting tourists and strengthening destinations’ brands. Therefore, this study can bridge the abovementioned research gaps by testing a proposed model in different cultural contexts and providing empirical evidence based on the stimulus–organism–response (S-O-R) model [17].

This study aims to examine the relationships between wellness motivation, engagement, satisfaction, and destination loyalty among wellness tourists. It also aims to examine the mediating effects of two engagement factors, experiential and reflective engagement, between wellness motivation and positive outcomes. This study tests a second-order construct of wellness motivation and can provide a theoretical rationale for a higher-order construct that includes multidimensions of wellness motivation. The results can contribute to highlighting the role of wellness motivations, cognitive and emotional aspects of engagement that influence wellness tourists’ experiences. This study can provide meaningful implications for maximizing positive behavioral intentions and the sustainable development of destination management, facilitating regional revitalization and strengthening a destination’s identity and attractions.

## 2. Literature Review

### 2.1. Wellness Tourism and Wellness Travel Motivation

The concept of wellness was proposed by Dunn [18], and various dimensions of wellness have been suggested by many scholars [5,6,13]. Previous research has developed various dimensions of wellness tourism and highlights the multifaceted aspects of wellness tourism on tourists’ health, such as physical aspects, as well as psychological, intellectual, emotional, social, and spiritual dimensions [5,6,18,19]. Many studies on wellness tourism have proposed multidimensional aspects of wellness tourism [5,6,7,8,13,20,21,22,23]. There is, however, no consensus on the motivation behind wellness tourism.

Research on wellness tourism contributes to an understanding of what motivates tourists’ decisions and how travel experiences influence their health and well-being [13,21]. Wellness is interconnected with multiple dimensions [19,24]. Wellness tourists participate in various activities related to health such as physical exercise, therapy, massages, spas, beauty treatments, nature-related activities, yoga, meditation, and so on [5,6,8,25,26,27]. Damijanić [5] proposed wellness motivation factors that are related to a healthy lifestyle and promote health conditions during an individual’s travel. Chi et al. [21] suggested that the physical, mental, and environmental wellness dimensions are important in wellness hotels.

Many researchers adopted the push and pull motivation theory [28,29] and demonstrated the effects of wellness motivation on tourists’ wellness travel behavior [8]. The adoption of push and pull motivations in previous research has been useful, and different combinations of push motivations, such as relaxation and novelty, and pull motivations, such as natural environments, landscapes, entertainment, and recreation, were employed [5,6,7,8,12,13]. Many studies on wellness tourism motivation have classified wellness tourists into different typologies and demonstrated their heterogeneity [5,6].

Physical motivation refers to a driving motivation to enhance one’s physical health and actively manage body fitness, physical appearance, and various physical aspects such as weight management through the limitation of high-calorie food, physical strength training, and looking after one’s physical well-being [5]. Physical motivation, physical fitness, and physical beauty are considered some of the most important wellness motivations [7,8,26,30]. Physical motivation is associated with an individual’s recognition of the importance of physical health to enhance their quality of life [5].

Transcendence refers to a profound understanding of the inner realms of the self and one’s life [5]. Moreover, through meditation, an individual can connect with one’s inner self, gain insights into themselves, and foster a sense of inner peace and spiritual experience [8,15].

Relaxation refers to resting to refresh the mind and body in order to increase rejuvenation and reduce stress and tension [8]. Relaxation helps individuals to recharge, refresh, and facilitate positive thinking and a positive state of mind. In addition, creating time for relaxation can increase one’s sense of control and mindfulness [7,31,32]. Previous research revealed that relaxation was the most influential motivation factor that led to wellness travel and behavioral intentions [15].

Social motivation is a force that drives individuals to desire connections with friends, family, and people with whom they have a meaningful relationship [5]. Social motivation is related to the creation of interpersonal relationships and the fulfillment derived from being together and enjoying life with family, friends, and significant others [7]. Social interaction provides emotional support, fosters social connections, and builds meaningful social bonds with like-minded individuals [12].

Self-esteem refers to the ability to find strength of the inner self and positive self-perception [13,15]. Individuals with high self-esteem can face difficult situations and cope with negative emotions. They also tend to find positive aspects and opportunities to overcome unfavorable circumstances with resilience and move in a positive direction in the future [15]. Travel experiences can enhance a sense of accomplishment and competence and provide an opportunity to elevate self-esteem [7].

Escape is the motivation to escape from daily concerns and seek rejuvenation by distancing from stress, worries, and problems [15,33,34]. When traveling, natural environments such as mountains, beaches, and rural areas serve as a temporary escape from stress and worries and create distance from people’s daily lives in urban environments, which comprise noisiness and crowdedness [32].

### 2.2. Wellness Motivation and Engagement

Previous research proposed that there are two aspects of engagement, namely experiential and reflective engagement [14]. Experiential engagement refers to forming emotional bonds with animals and environments in nature, encountering surprises, and experiencing a sense of wonder, contributing to an immersive and enjoyable overall experience [14,15]. Reflective engagement refers to the processing of experiences, which involves both cognitive and/or affective aspects. It also encompasses tourists’ memories and responses while acquiring new ideas in connection with learning outcomes [14]. Previous research has used two aspects of engagement and demonstrated the effects of experiential and reflective engagement in various travel contexts such as responsible tourism [35], nature-based tourism [14], wellness tourism [15], island tourism [36], and wildlife and ecotourism [16,36,37]. Experiential engagement enables the active exploration of attractions, involvement in travel activities, and interactive experiences with local environments. In contrast, reflective engagement is connected to the process of deriving meaning from travel experiences, wherein tourists engage in looking back on, evaluating, and interpreting their journeys retrospectively [35].

Tourists tend to engage in reflective contemplation on their actions, and they are encouraged to reflect on their actions during travel experiences, leading to positive changes in their behaviors aligned with new insights, pro-environmental behaviors, responsible travel behaviors, and well-being priorities [14,16,38,39]. Specifically, Lee, Jan, and Huang [40] revealed that experiential engagement had a positive effect on environmentally responsible behavior, and it was noted that tourists may feel positive emotions through recreational activities and prefer to enjoy natural environments and engage in general pro-environmental actions. Recently, previous research [14,16] demonstrated that ecotourism guiding interpretation programs and ecological education are positively associated with ecotourism behavior mediated by reflective engagement. Moreover, reflective engagement increases the effects of environmental information at destinations on tourists’ environmental concerns and environmentally friendly behaviors.

### 2.3. Motivation and Engagement

Previous research empirically demonstrated the causal relationships between motivation and engagement [14,15,41,42]. Ballantyne, Packer, and Falk [14] found that experiential and reflective engagement positively influences environmentally friendly behaviors. The results indicated that tourists’ wellness travel experiences elicit cognitive and affective responses that show a mediating effect between motivation and learning outcomes. Kim, Chiang, and Tang [15] examined the effects of four wellness travel motivations on experiential and reflective engagement in Taiwan. Their study highlighted the positive effects of wellness motivation on two engagement factors. So, Li, and Kim [43] conducted a systematic literature review and found that motivation leads to engagement at cultural and heritage sites and travel destinations for special-interest destinations.

**H1.**  *Wellness motivation has a positive effect on experiential engagement.*

**H2.**  *Wellness motivation has a positive effect on reflective engagement.*

### 2.4. The Relationship between Engagement, Satisfaction, and Loyalty

Satisfaction refers to a positive emotional state after evaluating received services or performance [44]. Loyalty refers to a deep commitment towards purchasing a favored product or service repeatedly ([45], p. 392). Engagement has emerged as a pivotal focus in previous research [46]. Understanding the positive outcomes of engagement is essential for developing destination marketing strategies and designing destination programs that fit in destination resources and attractions [47]. Moreover, the results can provide effective strategies for improving experiential quality and stimulating destination experiences that lead to active engagement [48].

Previous research identified satisfaction and destination loyalty as positive consequences after experiencing destination attractions among tourists [49]. Identifying antecedents of positive outcomes such as satisfaction and destination is crucial for providing memorable travel experiences and maximizing positive outcomes [50]. Emotional and cognitive engagement among tourists enhances their overall satisfaction and tourists’ intentions to revisit [47,48]. Engaging in customer experiences facilitates customer satisfaction, continuous engagement, and positive WOM [51].

Previous research examined the dynamic relationship between engagement, satisfaction, and loyalty [47,48,52,53,54]. As there are few empirical studies, research on the effects of engagement on satisfaction and behavioral intentions is necessary. Extant studies demonstrated positive effects between engagement, satisfaction, and loyalty [42]. Specifically, previous research found partial mediating effects between engagement, satisfaction, and destination loyalty [47]. Destination brand engagement positively affects tourists’ destination recommendation intentions and intentions to revisit [41,43,46,55,56]. Therefore, we propose that two engagement constructs positively influence satisfaction and destination loyalty.

**H3.**  *Reflective engagement has a positive effect on satisfaction.*

**H4.**  *Experiential engagement has a positive effect on satisfaction.*

**H5.**  *Reflective engagement has a positive effect on destination loyalty.*

**H6.**  *Experiential engagement has a positive effect on destination loyalty.*

### 2.5. Satisfaction and Destination Loyalty

**Figure 1:**
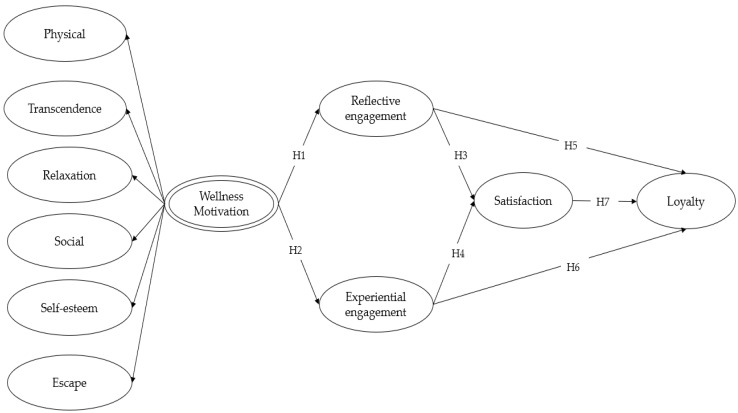
The proposed model.

Many scholars examined the relationship between satisfaction and loyalty and identified the positive relationship between satisfaction and destination loyalty [16,47,48,49,57,58]. Satisfied tourists tend to show their intention to revisit and share positive experiences and stories with others [59]. Kumar, Panda, and Adhikari [48] provided empirical evidence of the effects of destination experience and engagement on satisfaction among tourists, advocacy behaviors such as loyalty and recommendation, and positive WOM. Recently, Bagheri and colleagues [60] examined the relationships between tourists’ experience, well-being, satisfaction, and loyalty. Their study found that satisfaction positively affected behavioral intention (Figure 1).

**H7.**  *Satisfaction has a positive effect on destination loyalty.*

## 3. Method

### 3.1. Research Design and Setting

This study used a cross-sectional design conducted in Korea, focusing on domestic tourists who engaged in wellness tourism across the nation. The selection of this study setting was influenced by the post COVID-19 surge in interest in well-being and health and within the population of nature-based tourism [3,4]. According to KOCIS [4], despite the severe blow dealt to the tourist market by the pandemic, the wellness and medical tourism industry quickly rebounded, recording a remarkable 70% growth in 2022. Tourists are increasingly pursuing wellness experiences, which include spa and beauty treatments, the use of traditional Korean medicine, visits to advanced health and medical clinics, and the consumption of health-focused foods and teas [2]. This study targeted domestic tourists.

### 3.2. Participants

To establish eligibility criteria for respondents, this study employed three screening questions. Participants were considered eligible if they: 1) were aged above 18 years, and 2) reported experiencing wellness tourism within the past year. Respondents in this study setting had engaged in wellness tourism during 2022 and 2023, and 3 had shared their wellness tourism travel experiences on their personal blogs on Never Blog, which is one of the largest and popular platforms in Korea. Approximately, a total of 9130 bloggers were identified. A weblink for the questionnaire was sent to individuals who posted about their travels and included wellness-related keywords in their writing. A purposive sampling approach was applied for survey distribution to potential respondents who had participated in wellness travel.

### 3.3. Variables

In initiating the data collection process, a questionnaire was developed. The questionnaire was composed of three sections. The first section included a brief explanation of the project, participants’ agreement to take part in the survey, screening questions, and travel characteristics. The second section included measurement items of the proposed model, such as wellness motivation, engagement, satisfaction, and destination loyalty. The third section included demographic characteristics. On the first page of the questionnaire, a brief explanation of the project was illustrated.

Wellness motivation consists of six constructs, namely physical motivation, transcendence, relaxation, novelty, social motivation, and escape [12,13,61]. A total of 27 items of wellness motivation were tested, and 12 items were excluded. A total of 11 items of engagement were tested, which consists of two constructs, namely experiential and reflective engagement [14,15]. A total of six items were excluded. A total of three items of overall satisfaction [45] were used. A total of three items of destination loyalty [45] were used. The items were measured by using a seven-point Likert scale anchored from (1) strongly disagree to (7) strongly agree.

### 3.4. Common Method Bias (CMB)

To reduce CMB, face and content validities were verified. Criterion validity was confirmed. To refine the developed questionnaire, a pilot test was performed with experts in hospitality and tourism and graduate students majoring in hospitality and tourism. The participants of the pilot test were asked to find any mistakes, comment on areas for improvement, and verify the readability of the measurement items. After discussing the flow of the questionnaire, the final version was created on an online survey platform.

### 3.5. Sample Size

To determine the statistical power of the sample size, G*Power software (program version 3.1.9.7.) was used, with a significance level of α = 0.05 and power = 0.95. The total sample size was calculated at 146 samples. Subsequently, additional data were collected for further analysis. All participants were asked to complete all questions before submission and received a USD 2-value online coupon. A total of 342 participants completed the questionnaire between June and August 2023. This study focused on domestic tourists in South Korea, and all participants were Korean. The surveys filled out by respondents who did not answer carefully were removed. A total of 319 respondents were used.

### 3.6. Statistical Methods

To analyze the proposed model, descriptive analysis, exploratory factor analysis, and confirmatory factor analysis methods were used. The structural equation modeling (SEM) was conducted using STATA 18.0.

## 4. Results

### 4.1. Demographic Characteristics

Information regarding demographic characteristics is presented in Table 1. Various age groups were included (M = 38). Regarding gender, more females (n = 252, 79%) participated than males (67, 21.0%). This trend was also noted in previous research [8]. This could be due to gender-related influences on wellness tourism-related behaviors and preferences. Approximately 23.2% of the participants were in the age group of between 30 and 34 years old (n = 74), followed by those aged between 35 and 39 (n = 70, 21.9%) years and 45 years and over (n = 66, 20.6%). Approximately 54.2% reported that they were married (n = 173, 54.2%), and 45.1% were single. In terms of education level, 66.5% reported that they were either currently pursing or had received a bachelor’s degree (n = 212, 66.5%). Regarding work, approximately half of the participants were in a full-time position (n = 177, 55.5%), and 12.5% reported that they were self-employed. Regarding household income, the monthly income level varied, and 30.1% reported that they earned from 2,000,000 to less than 4,000,000 (n = 96). As noted in Table 1, USD 1 dollar equals KRW 1330.

### 4.2. Factor Analysis

This study employed two factor analysis approaches, namely exploratory factor analysis (EFA) and confirmatory factor analysis (CFA) [62]. These methods were utilized to reveal latent factors within the suggested conceptual model. The application of two-step factor analysis approaches allowed for the confirmation of measurement validation in the model.

First, the EFA results revealed multiple underlying factors, with a total of 15 items of wellness motivation. A total of 15 items of wellness travel motivation were found in six dimensions. After deleting an overloading measurement item, the results indicated that the Kaiser–Meyer–Olkin (KMO) value was 0.806 and Bartlett’s test (χ2 = 2169.470, *df* =105, *p*-value < 0.000) fit the criteria. The factor loadings ranged from 0.712 to 0.880. Six factors of wellness travel motivation were identified. Second, the EFA results for engagement indicated a good fit of KMO (0.705) and Bartlett’s test (χ2 = 418.754, *df* =10, *p*-value < 0.000). The experiential and reflective engagement dimensions were identified from five items. The factor loadings ranged from 0.708 to 0.883. Third, the EFA results for satisfaction indicated a good fit of KMO (0.751) and Bartlett’s test (χ2 = 720.955, *df* =3, *p*-value < 0.000). The factor loadings ranged from 0.917 to 0.945. Finally, the EFA results of loyalty indicated a good fit of KMO (0.747) and Bartlett’s test (χ2 = 760.047, *df* =3, *p*-value < 0.000). The factor loadings ranged from 0.913 to 0.950.

First, after conducting EFA, the results indicated that the Kaiser–Meyer–Olkin (KMO) values were all above 0.7, and Bartlett’s tests are all significant. The factor loadings of wellness motivation, engagement, satisfaction, and loyalty ranged from 0.708 to 950. Cronbach’s alpha ranged from 0.712 to 0.925. In terms of Cronbach’s alpha, the values aligned with the recommended standards [63].

The results substantiated discriminant validity, convergent validity, and compositive validity, in accordance with the recommended criteria suggested by Byrne (2013) [64]. The standardized regression coefficients as estimated by the CFA are provided in Table 2. Table 2 and 3 present the results providing the recommended model fit indices. The results in Table 2 and Table 3 demonstrate favorable model fit indices, with all values reaching the recommended criteria [64,65]. Within Table 3, the composite reliability (CR) values range from 0.816 to 0.935. The AVE values consistently surpassed 0.50, with the AVE of experiential engagement and the second order of wellness motivation being in close proximity to the 0.50 threshold. Both the CRs and Cronbach’s alpha exceeded the recommended levels, affirming the reliability of the measurement items. Convergent validity and discriminant validity were confirmed through the application of two sequential factor analysis approaches [66].

### 4.3. Structural Model

The SEM results are described as presented in Table 4 and Table 5. The model fit indices presented satisfactory values (χ2 = 736.580, *df* = 286, χ2/*df* = 2.575, GFI = 0.862, CFI = 0.910, TLI = 0.898, RMSEA = 0.070, SRMR = 0.081) [67]. The results showed that six dimensions of wellness motivations were significantly associated with the second-order wellness motivation. Of the motivations, relaxation had the highest regression coefficient (β = 0.898, *p* < 0.001), followed by physical wellness (β = 0.596, *p* < 0.001), escape (β = 0.570, *p* < 0.001), social (β = 0.490, *p* < 0.001), transcendence (β = 0.469, *p* < 0.001), and self-esteem (β = 0.453, *p* < 0.001).

The results revealed that five hypotheses were statistically significant, as shown in Table 5. Specifically, wellness motivation was positively associated with reflective engagement (β = 0.605, *p* < 0.001). Wellness motivation was positively associated with experiential engagement (β = 0.833, *p* < 0.001). Reflective engagement had a positive effect on satisfaction (β = 0.169, *p* < 0.01). Experiential engagement had a positive effect on satisfaction (β = 0.647, *p* < 0.001).

**Figure 2:**
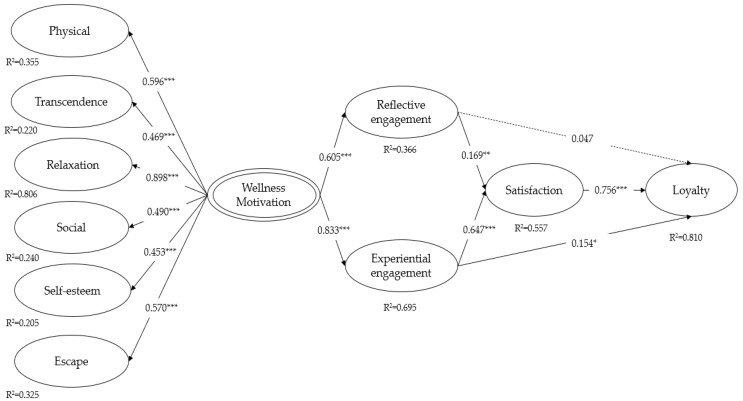
The results of the model. Note: * *p* = < 0.05; ** *p* = < 0.01; *** *p* = < 0.001.

Reflective engagement did not have a positive effect on destination loyalty (β = 0.047, *p* = 0.297). Experiential engagement had a positive effect on destination loyalty (β = 0.154, *p* < 0.05). Satisfaction had a positive effect on wellness destination loyalty (β = 0.756, *p* < 0.001). The regression coefficients of the proposed model are shown in Figure 2. The R-squared value for physical motivation was R^2^ = 0.355; for transcendence, R^2^ = 0.220; for relaxation, R^2^ = 0.806; for social motivation, R^2^ = 0.240; for self-esteem, R^2^ = 0.205; and for escape, R^2^ = 0.325. Regarding engagement, the R-squared value for experiential engagement was 0.695. The R-squared value for reflective engagement was 0.366. The R-squared value for satisfaction was 0.557, and the R-squared value for loyalty was 0.811.

### 4.4. The SEM Effects of the Model

The SEM effects are presented in Table 6. Four indirect effects were all statistically significant. Specifically, wellness motivation had a statistically significant indirect effect on satisfaction (β = 0.489, *p* < 0.001). The three dimensions of wellness motivation (β = 0.642, *p* < 0.001), experiential engagement (β = 0.489, *p* < 0.001), and reflective engagement (β = 0.128, *p* < 0.05) had statistically significant indirect effects on loyalty.

## 5. Discussion

This study proposed a conceptual model for understanding the relationship between wellness motivation, experiential and reflective engagement, satisfaction, and destination loyalty. The results revealed that wellness motivation is an important antecedent of engagement, satisfying travel, and the intent to revisit and provide recommendations. In addition, travelers’ engagement and satisfaction had a mediating effect on the relationship between wellness motivation and destination loyalty.

### 5.1. Theoretical Implications

The results provide various theoretical implications. First, this study employed a second-order factor analysis to examine the multiple components of wellness motivation. As noted by previous research on wellness travel motivation [5,13], wellness motivation is composed of six wellness motivation components, namely physical motivation, transcendence, relaxation, social motivation, self-esteem, and escape, each representing first-order factors. These motivations focus on push motivation factors of wellness travel motivations. This study not only identified six components of wellness motivation as first-order factors, but also conducted a second-order factor analysis to reveal a higher-level construct named wellness motivation. The results contribute to expanding theoretical frameworks. Consistent with previous research [7,15,30], relaxation is one of the most important motivations among wellness tourists. The pursuit of relaxation emerges as a primary motivation among wellness tourists. The results indicate that wellness tourists prioritize and seek opportunities to engage in restorative and immersive activities. Unlike previous research [13,15], novelty was not included as a wellness motivation factor in this study.

Second, wellness motivation is positively associated with reflective and experiential engagement. The results confirmed that experiential and reflective engagement contribute to the identification of a multidimensional framework for engagement. Wellness motivation positively influences experiential engagement. Tourists engage actively when exposed to aesthetically enriching environments at travel destinations. Moreover, the presence of great scenery and enjoyable and pleasurable experiences can enhance experiential engagement by increasing satisfaction and travel experiences [68]. Wellness motivation is positively associated with reflective engagement. Wellness travel can provide opportunities for tourists to gain new ideas and insights. Moreover, wellness travel aligns with reflective engagement goals.

Consistent with previous research [47,48], engagement positively affects satisfaction and destination loyalty. The results showed that satisfaction was influenced by both experiential and reflective engagements. Experiential engagement had a higher effect on satisfaction than reflective engagement. Experiential engagement had a positive effect on destination loyalty. However, reflective engagement did not have a statistically significant effect on destination loyalty. Satisfaction positively affected destination loyalty. The results were consistent with previous research [16,47,48,49,57].

Finally, the results present a mediating effect on the relationship between wellness motivation, satisfaction, and destination loyalty. The results reveal that both experiential and reflective engagement act as mediators, influencing how wellness motivation is associated with satisfaction and destination loyalty. As a few studies have indicated [41], the results demonstrated that highly motivated tourists who actively participate in and reflect on their wellness travel experience show a higher level of satisfaction and destination loyalty.

### 5.2. Practical Implications

The results have several practical implications. The results suggest there are multiple dimensions to wellness motivation components. Tourists may have various motivations, such as relaxation, physical motivation, escape, social motivation, transcendence, and self-esteem. Wellness tourists can be motivated to visit travel destinations to maintain physical fitness, body and skin care, and good health. Moreover, they can engage in deep meditation, which helps them to understand themselves better, and they have an opportunity to engage in self-discovery and realize their potential and life goals. Marketing campaigns emphasizing relaxation and stress relief can attract tourists seeking peaceful gateways. Natural environments have a calming effect. Tourism businesses can integrate nature-based activities such as hiking, beach walks, and forest retreats into their offerings to tap into the relaxation component. The tourism industry can effectively cater to individuals motivated by relaxation, offering experiences and services that prioritize tranquility and well-being.

The results suggest that practitioners and DMOs need to actively design and develop experiential aspects of destination attractions for tourists that align with their wellness motivations. Tailored approaches and practitioners’ efforts to attract wellness tourists facilitate tourists’ engagement and improve tourists’ experiences. Collaborating with accommodation facilities and resorts that prioritize a calm ambiance, comfortable amenities, holistic healing experiences, and scenic views contributes to the relaxation and escape appeal for individuals seeking rejuvenation and refreshment.

It is important to broaden the range of wellness experiences available at travel destinations. Wellness travel attractions need to be well-maintained and easily accessible for the enjoyable and pleasurable experiential engagement of wellness tourists. Wellness travel can foster emotional connections and contribute to a more meaningful and reflective travel experience. Involving experiential engagement and having various opportunities to view inspiring scenery, experience enjoyable engagement, and have a pleasurable experience are positively linked to satisfaction. It is important to provide opportunities for tourists to reflect and gain new insights and offer a diverse array of engagement opportunities at wellness destinations to increase overall satisfaction and destination loyalty.

One of the goals among DMOs and practitioners is to increase destination loyalty. Satisfied and loyal tourists express positive impressions and recommend the destination to others, contributing to repeated visits to the destination and the dissemination of positive WOM online and offline.

Destination managers can strengthen tourists’ loyalty toward wellness destinations by prioritizing engaging and memorable experiences for tourists that align with wellness motivations. Creating programs to enhance experiential and reflective engagement is a key strategy for building relationships and personal connections with tourists. Marketers can tailor wellness destination campaigns by considering experiential and reflective engagement. Crafting engaging experiences can contribute to more meaningful and satisfying travel experiences and enhanced destination loyalty.

### 5.3. Limitations and Future Research Contributions

The results provide various implications; however, some limitations should be noted. First, the results may not be generalized. The data were collected by using a cross-sectional study design. Other temporal factors such as technological changes and seasonality can influence the relevance of the results to the research circumstances. Future research should replicate the proposed model for validation. Second, this study focused on domestic tourists. Diversity within tourists from different countries could be considered. Moreover, tourists from different cultural backgrounds and those with distinct characteristics (e.g., unique segments) can show various preferences, motivations, and behavioral intentions. Third, this study used a cross-sectional design, and a different research approach, such as qualitative research or longitudinal research, could be suggested. This study was limited from the examination of why people are motivated, engaged, and show behaviors, and changes over time could not be identified. The use of another methodological approach could extend our robust understanding of the relationships in the proposed conceptual model. Future research can explore dimensions suggested in the proposed model, exploring their contributions to the motivation processes and their effects on destination loyalty.

## 6. Conclusions

First-order wellness motivation was identified, and the utilization of a second-order factor analysis allowed us to gain an understanding of the inter-relation. This study provided empirical evidence and offered a deeper understanding of wellness tourists’ motivational processes using a proposed conceptual model. A second-order factor model encompassing six wellness motivation factors provided another approach for estimating wellness motivation components influencing engagement, satisfaction, and behavioral intentions. Wellness motivation leads to engagement in activities and wellness environments that evoke emotional and cognitive responses. Wellness tourists have opportunities to reflect and gain new ideas and sources of inspiration, which encourage them to enjoy beautiful scenery and have pleasurable experiences with family, friends, and travel companions. The proposed model demonstrated that wellness motivation and two forms of engagement enhance overall satisfaction and destination loyalty. Wellness tourists with high satisfaction show a higher level of destination loyalty.

## Figures and Tables

**Table 1:** Demographic characteristics.

Variable	Category	n	%
Gender	Male	67	21.0
	Female	252	79.0
Age (M = 38)	18–24	16	5.0
	25–29	48	15.0
	30–34	74	23.2
	35–39	70	21.9
	40–44	45	14.1
	45 and over	66	20.6
Marital status	Single	144	45.1
	Married	173	54.2
	Other	2	0.6
Education level status	High school	19	6.0
	Associate	34	10.7
	Bachelor’s degree	212	66.5
	Post-graduate	54	16.9
Occupation	Full-time	177	55.5
	Part-time	16	5.5
	Self-employment	40	12.5
	Unemployed	7	2.2
	Student	7	2.2
	Other	72	22.6
Monthly household income (KRW)	Under KRW 2,000,000	14	4.4
	2,000,000–less than 4,000,000	96	30.1
	4,000,000–less than 6,000,000	70	21.9
	6,000,000–less than 8,000,000	60	18.8
	8,000,000–less than 10,000,000	47	14.7
	10,000,000 and over	32	10.0

Note: USD 1 = KRW 1330.

**Table 2:** The measurement items.

	Item	Factor Loading	M (SD)	CR	AVE
(1) Physical	Physical1	0.684	5.349 (1.070)	0.856	0.668
	Physical4	0.869			
	Physical5	0.884			
(2) Transcendence	Transcendence1	0.857	4.756 (1.160)	0.852	0.659
	Transcendence2	0.720			
	Transcendence3	0.851			
(3) Relaxation	Relax1	0.720	6.163 (0.731)	0.774	0.533
	Relax3	0.752			
	Relax_indulgence3	0.718			
(4) Social	Social1	0.716	5.010 (1.305)	0.716	0.557
	Social2	0.776			
(5) Self-esteem	Selfesteem2	0.842	4.594 (1.472)	0.805	0.674
	Selfesteem3	0.799			
(6) Escape	Escape4	0.913	5.408 (1.270)	0.839	0.723
	Escape5	0.783			
(7) Experiential engagement	Experiential1	0.748	6.007 (0.724)	0.737	0.484
	Experiential3	0.639			
	Experiential5	0.696			
(8) Reflective engagement	Reflective1	0.654	5.063 (1.100)	0.763	0.622
	Reflective2	0.904			
(9) Satisfaction	Satisfaction1	0.862	5.985 (0.826)	0.923	0.799
	Satisfaction2	0.918			
	Satisfaction3	0.900			
(10) Loyalty	Loyalty1	0.862	6.003 (0.871)	0.928	0.811
	Loyalty2	0.935			
	Loyalty3	0.903			

**Table 3:** The CFA results from the second-order model.

	WM	EE	RE	SAT	L
Wellness motivation (WM)	**0.700**				
Experiential engagement (EE)	0.288	**0.696**			
Reflective engagement (RE)	0.284	0.272	**0.789**		
Satisfaction (SAT)	0.269	0.366	0.334	**0.894**	
Loyalty (L)	0.278	0.374	0.346	0.566	**0.901**

Goodness-of-fit of the model. Note: χ2 (283) = 736.264, *p* < 0.001; χ2/df = 2.603; GFI = 0.862; CFI = 0.910; TLI = 0.896; RMSEA = 0.071; SRMR = 0.081. The diagonal values are the square root of AVE.

**Table 4:** The SEM results of the second order of wellness motivation.

			Coef.	z
Wellness motivation	→	Physical	0.596 ***	11.81
Wellness motivation	→	Transcendence	0.469 ***	7.56
Wellness motivation	→	Relaxation	0.898 ***	21.15
Wellness motivation	→	Social	0.490 ***	8.02
Wellness motivation	→	Self-esteem	0.453 ***	7.25
Wellness motivation	→	Escape	0.570 ***	10.50

Note: *** *p* = < 0.001.

**Table 5:** The SEM results in the proposed model.

				Coef.	z	Support
H1	Wellness motivation	→	Reflective engagement	0.605 ***	9.92	Yes
H2	Wellness motivation	→	Experiential engagement	0.833 ***	19.59	Yes
H3	Reflective engagement	→	Satisfaction	0.169 **	2.77	Yes
H4	Experiential engagement	→	Satisfaction	0.647 ***	12.04	Yes
H5	Reflective engagement	→	Loyalty	0.047	1.04	No
H6	Experiential engagement	→	Loyalty	0.154 *	2.07	Yes
H7	Satisfaction	→	Loyalty	0.756 ***	14.57	Yes

Note: * *p* = < 0.05; ** *p* = < 0.01; *** *p* = < 0.001.

**Table 6:** The SEM effects.

			Direct		Indirect		Total	
			Coef.	z	Coef.	z	Coef.	z
Wellness motivation	→	Experiential engagement	0.833 ***	6.55			0.833 ***	6.55
Wellness motivation	→	Reflective engagement	0.605 ***	5.39			0.605 ***	5.36
Wellness motivation	→	Satisfaction			0.489 ***	7.29	0.641 ***	6.68
Experiential engagement	→	Satisfaction	0.647 ***	8.72			0.647 ***	8.72
Reflective engagement	→	Satisfaction	0.169 **	2.63			0.169 **	2.63
Wellness motivation	→	Loyalty			0.642 ***	6.72	0.642 ***	6.72
Experiential engagement	→	Loyalty	0.154 *	2.46	0.489 ***	7.29	0.643 ***	8.76
Reflective engagement	→	Loyalty	0.047	1.11	0.128 *	2.58	0.175 **	2.79
Satisfaction	→	Loyalty	0.756 ***	11.30			0.756 ***	11.30

Note: * *p* = < 0.05; ** *p* = < 0.01; *** *p* = < 0.001.

## Data Availability

Data are available upon request from the authors.
